# Emerging role of interleukin‐13 in cardiovascular diseases: A ray of hope

**DOI:** 10.1111/jcmm.16566

**Published:** 2021-05-04

**Authors:** Ningjing Qian, Ying Gao, Jian’an Wang, Yaping Wang

**Affiliations:** ^1^ Department of Cardiology The Second Affiliated Hospital Zhejiang University School of Medicine Hangzhou China; ^2^ Cardiovascular Key Lab of Zhejiang Province Hangzhou China

**Keywords:** cardiovascular diseases, immunotherapy, inflammation, interleukin‐13

## Abstract

Despite the great progress made in the treatment for cardiovascular diseases (CVDs), the morbidity and mortality of CVDs remains high due to the lack of effective treatment strategy. Inflammation is a central pathophysiological feature of the heart in response to both acute and chronic injury, while the molecular basis and underlying mechanisms remains obscure. Interleukin (IL)‐13, a pro‐inflammatory cytokine, has been known as a critical mediator in allergy and asthma. Recent studies appraise the role of IL‐13 in CVDs, revealing that IL‐13 is not only involved in more obvious cardiac inflammatory diseases such as myocarditis but also relevant to acute or chronic CVDs of other origins, such as myocardial infarction and heart failure. The goal of this review is to summarize the advancement in our knowledge of the regulations and functions of IL‐13 in CVDs and to discuss the possible mechanisms of IL‐13 involved in CVDs. We highlight that IL‐13 may be a promising target for immunotherapy in CVDs.

## 1 INTRODUCTION

Cardiovascular diseases (CVDs) remain an important public health problem worldwide, causing large disease and economic burden. Despite the development of new drug and non‐drug therapy, in the recent few decades, the morbidity and mortality keep disturbingly high.^1^ Accumulating evidence has suggested that inflammation plays a crucial role in the development and progression of CVDs and may be potential therapeutic targets.^2^, ^3^ In 1990, Levine et al^4^ firstly reported the association of inflammation with the pathogenesis of CVDs by revealing the elevated level of tumour necrosis factor (TNF) in patients with chronic heart failure. Several markers of inflammation including interleukin (IL)‐6, IL‐1β, C‐reactive protein (CRP) are subsequently observed with increased levels in patients with CVDs.^3^, ^5^ Meanwhile, findings have shown that risks of CVDs are significantly higher in patients with systemic chronic inflammation such as periodontitis,^6^ systemic lupus erythematosus^7^ and atopic dermatitis.^8^ Recently, the Canakinumab Anti‐inflammatory Thrombosis Outcome Study (CANTOS) indicates the significantly lower rate of recurrent cardiovascular events, reduced heart failure‐related hospitalizations and mortality with the treatment of a monoclonal antibody targeting IL‐1β, which further highlights the crucial role of inflammation in CVDs.^9^, ^10^

Cytokines are essential mediators of inflammation regulating a set of biological mechanisms through autocrine, paracrine or endocrine.^11^ IL‐13, one of the powerful cytokines with broad functions, is widely expressed in most tissues, such as heart, lung, liver and skin.^12^ IL‐13 is recognized to be engaged in regulating cell‐mediated immunity, modulating cell proliferation, growth and apoptosis and taking part in the genesis and development of a variety of diseases, including allergic asthma, chronic obstructive pulmonary disease, schistosomiasis, hepatic fibrosis and cancers.^13^, ^14^ Recently, IL‐13 is identified to be associated with CVDs. The current review shall familiarize readers with IL‐13 and its role in CVDs. And we also discuss the possibility of IL‐13 as a future target for treating CVDs.

## 2 EXPRESSION AND RECEPTORS OF IL‐13

**FIGURE 1:**
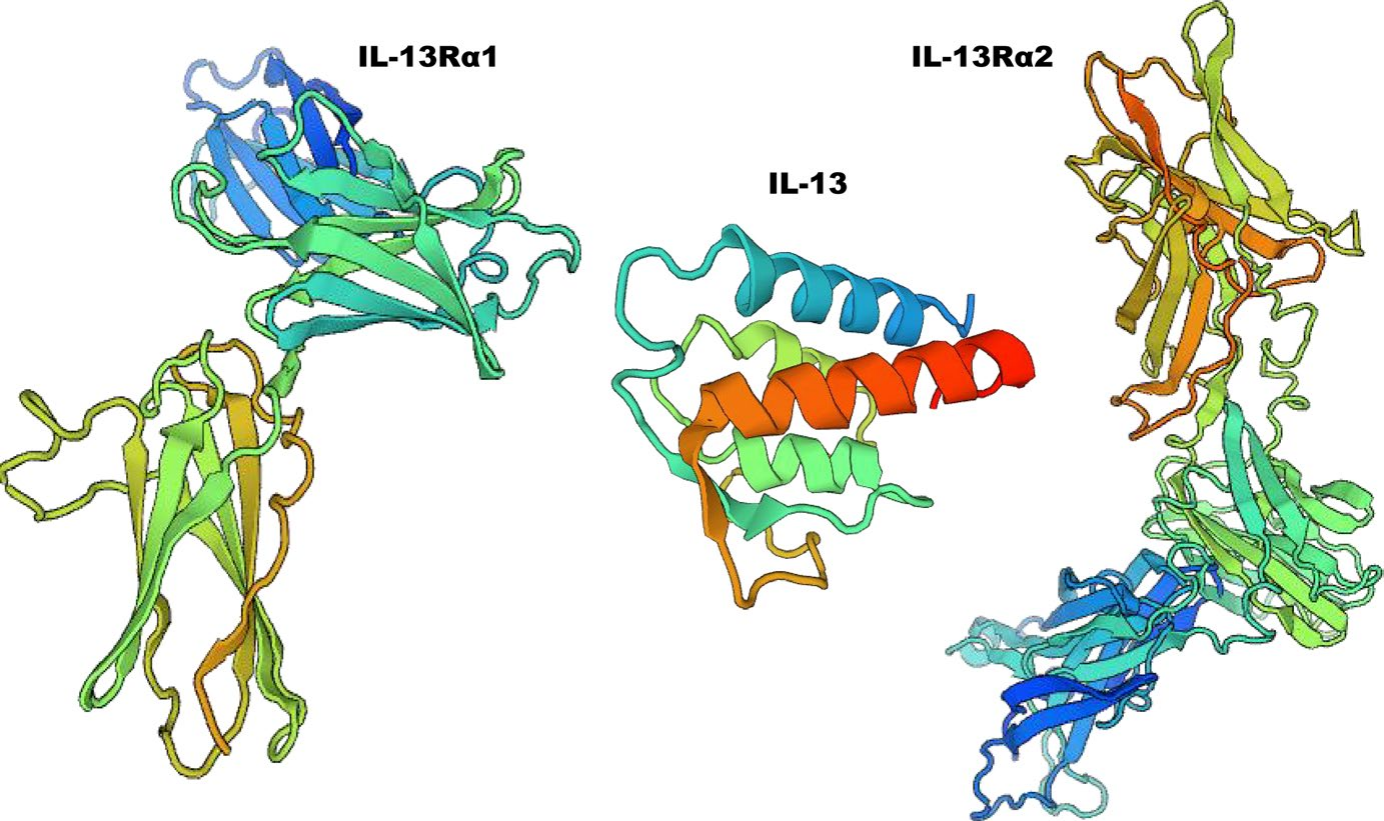
The three‐dimensional structure of IL‐13, IL‐13Rα1and IL‐13Rα2. SWISS‐MODEL (www.swissmodel.org) was used to view the graphic. IL‐13, interleukin‐13; IL‐13R, interleukin‐13 receptor

IL‐13 was originally reported as a Th2 type cytokine expressed by CD4+T cells.^15^ Recently, accumulating evidence demonstrates type 2 innate lymphoid cells (ILC2s) are a primary source of IL‐13.^16^ Prior studies indicate that ILCs have strong tissue tropism and ILC2s are dominantly present in tissues serving as barriers, such as lung, gut and skin, to early engage in immune responses by producing IL‐13.^17^, ^18^ That is also why the role of IL‐13 has been extra concerned in diseases such as asthma^15^ and atopic dermatitis.^8^ Recently, heart ILC2s are proven a predominant population of tissue‐resident ILCs with unique phenotypes, even greater than lung ILC2s.^19^ Like all ILCs, heart ILC2s are generated by foetal liver and seed tissues during foetal stage.^17^ And follow‐up studies suggest that the frequencies of heart ILC2s reach a peak at the age of 4 weeks after birth and then heart ILC2s maintain mostly by self‐renewal under physiological status. Under pathophysiological status, heart‐resident ILC2s rapidly respond to cardiac injury and show almost 7‐fold stronger ability of IL‐13 secretion compared with lung ILC2s.^19^ Actually, the secretion of IL‐13 could be triggered by infection, physical, chemical and metabolic noxious stimuli to drive type 2 immune responses while might be associated with age, lifestyle, social and physical factors as well.^20^ The heterodimerization of IL‐13 receptor α chain (IL‐13Rα)1 and IL‐4 receptor chain (IL‐4R) leads to the formation of the IL‐13 receptor (IL‐13R) and IL‐13 binds to IL‐13R on target cells to induce specific functions.^13^ And IL‐13Rα2 is identified to form a decoy receptor that suppresses IL‐13 induced immune response.^21^ The three‐dimensional structure of IL‐13, IL‐13Rα1 and IL‐13Rα2 is shown in Figure 1. The distribution of IL‐13R has been carefully investigated by Akaiwa et al^12^ in each tissue. IL‐13R is found localized in skin, nasal mucosa, bronchial, liver, heart and stomach while detectable in brain and bone marrow. Specifically, in heart IL‐13R is strongly expressed in cardiomyocytes, fibroblasts, vascular smooth cell and endothelial cells but not in intramuscular connective and epicardial adipose tissue.

## 3 ROLE OF IL‐13 IN CVDS

### 3.1 Role of IL‐13 in heart failure

Heart failure (HF) is the terminal manifestation of multiple CVDs. HF is a loss of balance between cardiac output and physiological demand, with structural, functional and metabolism alterations of hearts.^22^, ^23^, ^24^ Growing evidence suggests that IL‐13 plays a crucial role in the initiation and development of HF. Significantly elevated serum level of IL‐13 has been detected in patients with chronic HF.^25^ The study in ageing mice models shows an age‐dependent increasing trend in IL‐13 expression in hearts accompanied by progressive cardiac dysfunction.^26^ The expansion of non‐classical CD14^dim^CD16^+^ monocytic subset could partly account for the increased production of IL‐13.^25^ Histone acetyltransferases and histone deacetylases (HDAC) 11 inhibits IL‐13 transcription by modulating the initiator of IL‐13, transcription factor E4‐binding protein 4 (E4BP4) in CD4^+^ T cells in the heart. Lower expression of HDAC11 is found in hearts with HF, which attenuates its repression of IL‐13, thus enhances the transcription of IL‐13.^27^

Increased IL‐13 is implicated in age‐dependent cardiac fibrosis, collagen production and pathological cardiac remodelling in vivo in mice model and is proven to promote monocyte‐fibroblast transformation to mediate fibrosis in vitro.^26^ Studies in transgenic mice with IL‐13Rα1‐deficient have shown that these mice have less cardiomyocyte hypertrophy, collagen deposition and show resistance to fibrosis in response to chronic pressure overload. The activation of IL‐13Rα1 modulates expression of transforming growth factor (TGF) β, and tissue inhibitor of metalloproteinase (TIMP) 1 that regulates the accumulation of extracellular matrix (ECM), thereby promoting fibrosis via signal transducer and activator of transcription (STAT) 3/STAT6 signalling.^28^ The role of IL‐13 in metabolic reprogramming has provided brand new insights into its active involvement in HF progression. Imbalanced metabolism homeostasis of HF is characterized by alteration of substrate utilization. Generally, healthy hearts shift from fatty acids utilization to glycolysis based metabolism to meet energy demand under conditions such as overload while failing hearts lose this flexibility.^29^, ^30^ Knudsen et al have found that enhanced IL‐13 up‐regulates fatty acids utilization in mice muscle via IL‐13Rα1‐STAT3 axis, at the expense of higher oxygen consusmption and mitochondrial respiration.^31^ Knudsen et al note that IL‐13 acts as a positive role to improve exercise endurance, but for an overwhelmed heart, IL‐13 might be the final straw.

### 3.2 Role of IL‐13 in myocardial infarction

Ischaemic heart disease, principally myocardial infarction (MI), has been a major threat to public health worldwide.^32^ MI is a consequence of sudden ischaemia and hypoxia attack of myocardial tissue.^33^ At present, reperfusion therapy is widely and commonly applied in the treatment of MI in order to restore perfusion and oxygen supply early, reduce cardiac damage and improve prognosis. However, the reperfusion itself could subsequently cause another injury, which is known as ischaemia/reperfusion (I/R) injury.^33^, ^34^

Hofmann et al^35^ have clearly illustrated the dynamic expression of IL‐13 after MI in vivo in mice MI models and in cardiomyocytes in vitro. The level of IL‐13 is increased immediately in infarct zone, reaches a peak at 3 days after MI, declines thereafter until day 7, and then is increased again. The study conducted by Yuan et al^34^ demonstrates that the levels of IL‐13 in heart tissues as well as in the serum are up‐regulated at 7 days after I/R and remain significantly high at day 14 in mice model. However, compared to healthy individuals, patients with MI at acute phase have markedly lower serum levels of IL‐13.^36^ And patients with MI undergoing reperfusion therapy by primary angioplasty show a reduction of IL‐13 in the serum at 3 months, which is positively associated with deteriorated cardiac function.^37^ Thus, it is supposed that IL‐13 dynamically functions in MI and the following I/R injury.

IL‐13 is involved in cardiac remodelling at the very early phase of MI, which is evidenced by the significantly worse survival and decreased echocardiography parameters of IL‐13 deficiency mice within 7 days after MI. IL‐13 deficiency mice demonstrate insufficient monocyte/macrophage‐driven wound healing, disordered collagen deposition and collagenase activity during scar formation, which abolishes the cardio‐protective effects of IL‐13 in response to MI.^35^ The benefits of IL‐13 in the infarcted heart is confirmed by another study. It is suggested that IL‐13 secreted by human embryonic stem cell derived‐cardiovascular progenitor cells (hESC‐CVPCs) could modulate macrophage polarization via STAT6 phosphorylation to improve cardiomyocyte survival and promote angiogenesis during the early phase of MI.^38^ Recently, IL‐13 is reported to accelerate cardiac regeneration to promote cardiac repair even in adult MI mice model depending on hepcidin‐deficient macrophages.^39^

### 3.3 Role of IL‐13 in myocarditis

Myocarditis refers to as localized or diffuse inflammatory lesions of the myocardium, initiated by infectious factors, most commonly, viruses and non‐infectious factors, such as systemic autoimmune disease, medications and cardiotoxins.^40^ It is believed that IL‐13 consecutively contributes to regulating recruitment and differentiation of immune cells, chemokines secretion and fibrosis during the progression of myocarditis.^40^, ^41^, ^42^ IL‐13 deficiency mice develop into severe myocarditis in both coxsackievirus B3 (CVB3)‐induced model and experimental autoimmune myocarditis (EAM) model, manifested in decreased systolic and diastolic left ventricular functions and decreased survival.^41^ Instead, administration with IL‐13 could significantly ameliorate the heart inflammation and cardiac injury caused by CVB3 induced myocarditis.^43^ The majority of immune cells in response to myocarditis are macrophages^40^ and IL‐13 targets on enhancing M2 macrophage polarization to protect the heart against myocarditis.^41^, ^43^, ^44^ IL‐13 could decrease both T lymphocyte infiltration of the heart and activation of T lymphocytes in the spleen and influence the production of interferon (IFN)‐γ and IL‐17, which mediate the differentiation of macrophages or directly prevent from myocarditis.^41^, ^43^ Meanwhile, IL‐13 reduces the production of IL‐1 and IL‐18 by preventing lipopolysaccharide‐dependent caspase‐1 activity in monocytes, in which way ameliorates inflammation against EAM.^41^ Curcumin, derived from turmeric, has been proved beneficial against EAM by inducing M2 macrophage polarization. Mechanistically, curcumin up‐regulates the secretion of IL‐4 and IL‐13 to activate STAT6 pathway in macrophages and in turn, promotes macrophages differentiation. Notably, the absence of IL‐13 in EAM is associated with more fibrotic alteration although IL‐13 is generally considered as a promoter of fibrosis.^14^, ^41^ A possible explanation is the increased production of pro‐fibrogenic factors, such as IL‐1β, IL‐4, TGFβ1, and histamine due to the deficiency of IL‐13. Overall, IL‐13 may benefit in the prevention of EAM and viral myocarditis. Conversely, IL‐13 appears to act as a culprit in eosinophilic myocarditis. IL‐13 is suggested to account for the cardiac damage by inducing the expression of eotaxins CCL11 in cardiac fibroblasts and eotaxins CCL24 in macrophages to recruit eosinophils to the heart.^45^

### 3.4 Role of IL‐13 in dilated cardiomyopathy

Dilated cardiomyopathy (DCM) is characterized as left ventricular dilatation and contractile dysfunction. The major causes of DCM include genetic mutation, myocarditis and metabolic and endocrine disturbances.^46^ Persistence of inflammatory infiltration has been detected in patients with DCM.^46^, ^47^ The existence of inflammatory would impede the response and benefit of cardiac resynchronization therapy^48^ while amelioration of myocardial inflammation contributes to the improvement of cardiac function in DCM.^49^ Ohtsuka et al^50^ demonstrated that patients with DCM had increased serum levels of IL‐13. Integrated backscatter (IB), a method of quantitative echocardiographic techniques was further used to assess the correlation between IL‐13 and myocardial fibrosis in DCM. Higher calibrated IB, representing heavier myocardial fibrosis, were positively associated with increased serum levels of IL‐13, in company with LV enlargement in DCM. However, IL‐13 showed no significant correlations with markers of LV systolic function in patients with DCM. Hence, IL‐13 might be a potential predictor or therapy target in DCM.

### 3.5 Role of IL‐13 in valvular heart disease

The prevalence of valvular heart disease (VHD) including degenerative valve diseases and rheumatic heart diseases remains high. Especially, the prevalence of degenerative valve diseases rises dramatically among individuals over 65 years old due to the predominance of degenerative aetiologies.^51^ Few studies have reported the role of inflammation and immune response in VHD. As mentioned above, there is an age‐dependent increase in IL‐13 in heart.^26^ IL‐13 is probably associated with VHD, especially degenerative valve diseases. Rotter et al^52^ have demonstrated that patients with aortic valve diseases show much higher circulating IL‐13 levels, compared to healthy individuals. The correlation between IL‐13 and VHD has been evidenced by another study based on patients with aortic stenosis or aortic inefficiency or tricuspid inefficiency.^53^ It is suggested that much higher expression of IL‐13 in VHD patients with thicker epicardial adipose tissue (EAT) is positively associated with the up‐regulation of TGFβ, metalloproteinases (MMPs) and osteogenic genes. In other words, IL‐13 is related to fibrosis and calcification in VHD.^53^ Consistently, the study in patients with rheumatic heart disease observed higher serum levels of IL‐13 and meanwhile increased local collagen deposition in valves, suggesting IL‐13 might induce fibrosis in rheumatic heart disease as well.^54^ Taken together, IL‐13 is associated with the progression of VHD and might serve as a promising candidate for VHD prognostic factor, which needs further elucidation.

## 4 DISCUSSION

**FIGURE 2:**
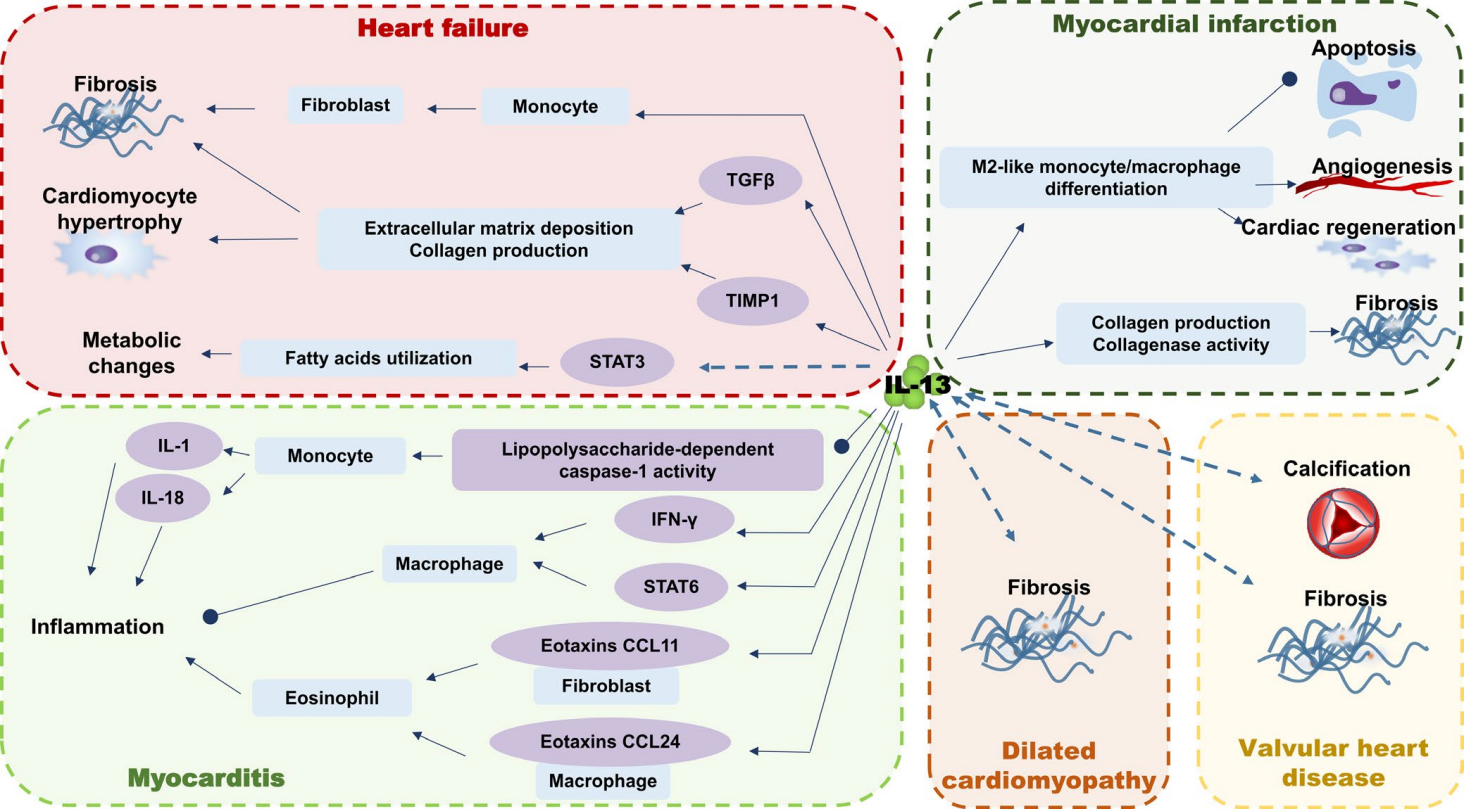
The function and mechanism of IL‐13 in cardiovascular diseases. IFN, interferon; IL‐13, interleukin‐13; STAT, signal transducer and activator of transcription; TGF, transforming growth factor; TIMP, transforming growth factor

Various studies in vitro and in vivo suggest that IL‐13 is widely involved in many CVDs while the role of IL‐13 can be a double‐edged sword, either cardio‐protective or deleterious properties under different conditions and course of diseases (Figure 2). IL‐13 acts positively to promote the development of heart during the early stage and facilitate the cardiac repair after MI.^35^, ^38^, ^39^, ^55^, ^56^ Interestingly, the expression level of IL‐13 experiences ‘up‐down‐up’ fluctuation after MI in mice model^35^ but patients with MI at acute phase are suggested with decreased levels of IL‐13.^36^ Thus, the alteration and function of IL‐13 during MI need further study. It is possible that IL‐13 is implicated in multiple pathophysiological processes and the time window could be critical for the potential intervention based on IL‐13. Prolonged synthesis of IL‐13 seems to be a risk factor of adverse outcomes in chronic CVDs, such as HF, largely due to the induction of fibrosis and adverse cardiac remodelling.^25^, ^26^, ^27^, ^28^ In addition, IL‐13 is newly reported driving metabolic reprogramming in muscle.^31^ Understanding the effect of IL‐13 on heart metabolism will shed light on possible new mechanisms for CVDs. The relationship between circulating levels and tissue levels of cytokines has also been investigated. Generally, circulating levels of IL‐13 go parallel with expression levels in the heart tissues, that is, circulating levels of IL‐13 could mirror the IL‐13 level patterns in the heart.^26^, ^34^ However, we cannot exclude the dissociation of systemic inflammation states and local secretion because studies have indicated the heart‐resident ILC2s and immune cells can be triggered and recruited locally to strongly produce IL‐13.^19^, ^27^ Furthermore, the role of IL‐13 in cardiac regeneration provides a new possible therapeutic strategy for CVDs. Due to the lack of regenerative capacity of adult human cardiomyocytes, it remains no specific treatment for CVDs that targets on restoring the missing and injured myocardium.^57^, ^58^ The barriers to heart regeneration have been such a difficult challenge for cardiac repair. Recently, IL‐13 has been identified as a critical regulator of cardiomyocyte cell cycle entry and differentiated state by O'Meara^59^ and his colleagues. Administration of IL‐13 could restore the regenerative capacity of cardiomyocytes and reverse the scar formation and cardiac dysfunction post‐neonatal injury in IL‐13 deficient mice.^55^, ^56^ By contrast, deficiency of IL‐13 restricts cardiomyocyte proliferation, induces compensatory cardiomyocyte hypertrophy in vitro and deletion of IL‐13 in neonatal mice results in cardiac dysplasia, impaired cardiac repair in vivo.^56^, ^59^

In a nutshell, the evidence so far suggests that IL‐13 is associated with cardiac fibrosis, cardiomyocyte proliferation, myocardial hypertrophy, recruitment and differentiation of immune cells and chemokines secretion in heart while the exact signalling pathways and underlying mechanisms are not fully understood. The recent CANTOS study targeting IL‐1β reminds us to consider novel treatment strategies based on immunotherapy.^3^, ^10^ The application of monoclonal antibodies against IL‐13, such as tralokinumab and dupilumab, has been investigated in the treatment of asthma.^15^ With abundant evidence regarding the association between IL‐13 and CVDs, there is scope for further research to reveal the role and underlying mechanisms of IL‐13 in CVDs to provide a solid foundation for future development of immunotherapy.

## CONFLICT OF INTEREST

The authors declared that there is no conflict of interest.

## AUTHOR CONTRIBUTIONS

**Ningjing Qian:** Writing‐original draft (lead); Writing‐review & editing (lead). **Ying Gao:** Writing‐original draft (supporting); Writing‐review & editing (supporting). **Jian'an Wang:** Supervision (equal); Writing‐review & editing (supporting). **Yaping Wang:** Funding acquisition (lead); Supervision (equal).

## Data Availability

The NCBI database accession number for the protein profile data of IL‐13, IL‐13Rα1 and IL‐13Rα2 reported in this article is (PBD ID:3LB6‐A, PBD ID: 3BPN, PBD ID:3LB6‐C). It was retrieved in FASTA format and uploaded to the online server SWISS‐MODEL (www.swissmodel.org) to build the models of target proteins.
